# Impact of Climate Changes on the Natural Prevalence of *Fusarium* Mycotoxins in Maize Harvested in Serbia and Croatia

**DOI:** 10.3390/foods12051002

**Published:** 2023-02-27

**Authors:** Elizabet Janić Hajnal, Jovana Kos, Bojana Radić, Mislav Anić, Radmila Radović, Nina Kudumija, Ana Vulić, Sanja Đekić, Jelka Pleadin

**Affiliations:** 1 Institute of Food Technology, University of Novi Sad, Bulevar cara Lazara 1, 21000 Novi Sad, Serbia; 2 Croatian Meteorological and Hydrological Service, Ravnice 48, 10000 Zagreb, Croatia; 3 Croatian Veterinary Institute, Laboratory for Analytical Chemistry, Savska Cesta 143, 10000 Zagreb, Croatia; 4 Faculty of Chemistry, Department of Analytical Chemistry, University of Belgrade, Sudentski trg 12-16, 11158 Belgrade, Serbia

**Keywords:** deoxynivalenol, fumonisins, zearalenone, T-2, HT-2, maize, Serbia, Croatia, weather conditions

## Abstract

Ongoing climate change may affect the susceptibility of plants to attacks by pathogenic, mostly mycotoxigenic fungi with a consequent increase in the presence of mycotoxins. *Fusarium* fungi represent one of the most important producers of mycotoxins, and are also important pathogens of agricultural crops. Therefore, the main aim of the study was to estimate the impact of weather parameters on the natural occurrence of *Fusarium* mycotoxins, such as deoxynivalenol (DON), fumonisins B_1_ and B_2_ (FUMs), zearalenone (ZEN), T-2, and HT-2 toxins (T-2/HT-2) in maize samples harvested from two neighboring countries, Serbia and Croatia, during a four-year production period (2018–2021). The frequency and contamination level of examined *Fusarium* mycotoxins varied by maize year of production and could be linked to weather conditions per investigated country. Among them, FUMs were found to be the most common contaminants (84–100%) of maize in both Serbia and Croatia. Additionally, a critical assessment of *Fusarium* mycotoxins occurrence in the last 10 years (2012–2021), for both Serbia and Croatia, was done. Results pointed out the highest contamination of maize from 2014, especially with DON and ZEN, in connection to extreme levels of precipitation observed in both Serbia and Croatia, whereas FUMs occurred with high prevalence from each of the ten investigated years.

## 1. Introduction

One of the major global food and feed safety issues is the presence of mycotoxigenic fungi and their secondary metabolites, mycotoxins. They reduce the quality of agricultural crop commodities and impact negatively on human and animal health [1,2,3]. The fungal genus *Fusarium* is one of the most important mycotoxigenic fungal genera in food and feed. Several species of the genus *Fusarium* can infect cereals, while the predominant species vary depending on the type of crop, geographical region, and environmental conditions [4,5]. *Fusarium* mycotoxins are produced by toxigenic fungi that naturally contaminate cereals, representing a source of serious concern in cereals and cereal-based products, which leads to harmful contamination of food and feed [6]. The main and most frequently occurring mycotoxins produced by *Fusarium* fungi in cereals are fumonisins, trichothecenes, and zearalenone (ZEN), followed by “emerging“ mycotoxins (such as moniliformin, fusaproliferin, enniatins, and beauvericin) and many other metabolites (such as bikaverin, culmorin, aurofusarin, fusaric acid, fusapyron, and butenolid) [7,8,9,10,11].

Fumonisins are mainly produced by *Fusarium* (*F.*) *verticillioides* and *F. proliferatum* [12] and represent one of the most important mycotoxins that occur in maize (*Zea mays*), particularly when maize is grown in warmer regions [7]. Recent studies on laboratory animals have clearly shown that prenatal exposure to fumonisins is highly detrimental to the offspring, which can be manifested by altered chemical coding of enteric neurons of the gut. Furthermore, the neurotoxic effect of fumonisins is also linked to the enteric nervous system of the fetus [13].

Fumonisin analogues are classified into four main groups: fumonisin A, B (FB), C, and P. However, among all analogs of fumonisins, FB_1_, FB_2_, and FB_3_ generally occur in nature most frequently, while FB_1_ usually occurs with the highest concentrations [14]. *F. langsethiae, F. poae, F. equiseti,* and *F. sporotrichoides* mainly produce trichothecenes type A (T-2 toxin, HT-2 toxins, neosolaniol, monoacetoxyscirpenol, and diacetoxyscirpenol), while *F. graminearum* and *F. culmorum* typically produce trichothecenes type B (mainly deoxynivalenol (DON), 3-acetyldeoxynivalenol, 15-acetyldeoxynivalenol, and nivalenol). DON is the most frequently detected mycotoxin among trichothecenes in cereals and cereal-based products [15,16]. Furthermore, fungal species of *F. culmorum, F. graminearum, F. equiseti, F. cerealis,* and *F. semitectum* are the most important producers of ZEN which very often infect cereals worldwide, mostly in temperate climates [9,17]. 

In general, at any stage of the food production process (pre-harvest, during harvesting, and storage), fungal production of mycotoxins can occur and expose consumers to the risk of contamination, either through food consumption directly or through feed indirectly [18,19]. The health risks, which are recognized worldwide, are associated with the consumption of contaminated cereal products with *Fusarium* mycotoxins [4,6,10,17]. These toxins have been associated with human esophageal cancer and birth defects, while animals manifest carcinogenic and genotoxic effects, and lead to reproductive disorders [4,20]. The International Agency for Research on Cancer (IARC) classifies FB_1_ into Group 2B as possibly carcinogenic to humans [21]. Furthermore, the frequent co-occurrence of *Fusarium* mycotoxins in maize raises the question of synergistic or additive actions in the manifestation of toxicity [14,22]. In order to protect human and animal exposure to mycotoxins, and also to reduce financial losses and improve international trade, numerous measures are needed at different levels. As a result, regulatory limits on significant levels of mycotoxins in food and feed are established by various authorities worldwide. One of them is the European Commission (EC) No. 1881/2006 [23], which has set maximum levels (MLs) for *Fusarium* mycotoxins such as FB_1_ and FB_2_, ZEN, and DON in different food and feed products, and Commission Recommendation 2013/165/EU on the presence of T-2 and HT-2 toxin in cereals and cereal products [24].

In addition to the direct health risk, the economic losses resulting from mycotoxicosis are enormous. Annual agricultural and industrial losses due to crops contaminated with mycotoxins are estimated in billions of dollars [10]. Among cereals, maize is one of the most susceptible to contamination by mycotoxigenic fungi and mycotoxins. Furthermore, maize is the leading cereal by production in the world and one of the most important agricultural products [25]. Its popularity as a crop is largely due to its versatile functionality as a food source for both humans and animals. In addition to contributing to human nutrition and animal feed, maize has great economic importance for Serbia and Croatia. With an average annual production of 7.4 million tons and an average annual export estimated at 2.7 million tons of maize per year (average data for the period 2018–2020), Serbia is one of the leading maize producers and exporters in Europe [25]. Furthermore, Serbia consumes about 4.7 million tons of maize annually, with the vast majority used for animal feed (4.3 million tons), and 0.4 million tons for food, seed, and industrial uses [26]. In Croatia, maize is the largest single crop with an average annual production of about 2.3 million tons and with average annual exports of about 0.9 million tons (average data for the period 2018–2020) [25]. The presence of toxigenic fungi and mycotoxins in maize is primarily influenced by climatic conditions, genetic factors, fungal activity, agronomic practices, and storage conditions [6,10]. Extreme weather conditions, as well as reduced annual environmental variability, can cause biotic and abiotic stress in plants [27]. It is thought that this can have a significant effect on the extent of *Fusarium* infection in plants [28]. Therefore, it is very important to take into account climatic conditions, as well as climate changes, when studying and analyzing mycotoxins, such as *Fusarium* mycotoxins.

Given that *Fusarium* mycotoxins represent a potential danger for primary agricultural production in Europe and the world, as well as a high risk to public health, it is necessary to continuously monitor them. Furthermore, increasingly significant changes in mycotoxin occurrence patterns in cereals due to extreme weather conditions, which are part of ongoing climate change, are of concern. Thus, the aim of this study was to examine and compare the influence of weather conditions on the occurrence and concentration of *Fusarium* mycotoxins, including DON, ZEN, FUMs (FB_1_ and FB_2_), T-2, and HT-2 in maize samples during a four-year period (2018–2021), and to discuss the results of these mycotoxin studies performed in the last 10 years (2012–2021) in two neighboring countries, Serbia and Croatia.

## 2. Materials and Methods

### 2.1. Samples

**Figure 1:**
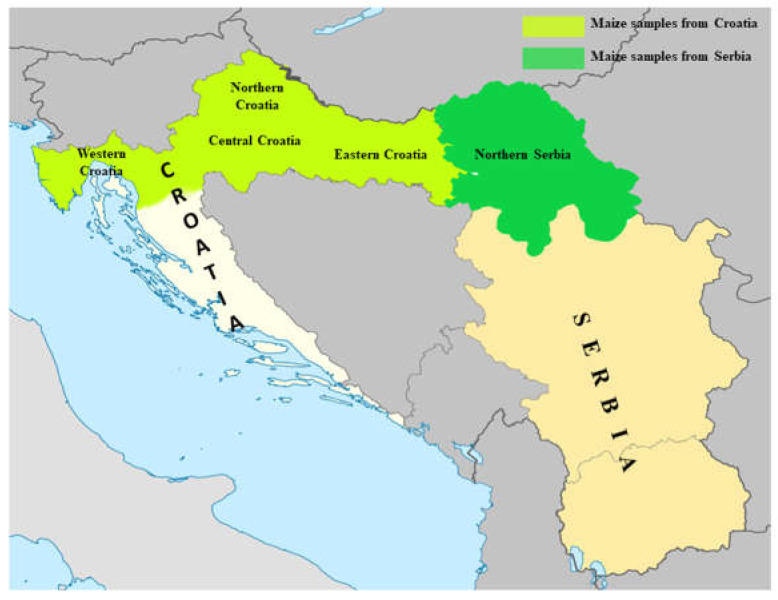
Regions of maize sampling in Serbia and Croatia.

Maize samples (n = 400) were randomly collected from the main maize producing areas in Northern Serbia (Autonomous Province of Vojvodina, latitude 45°18″ (N), longitude 20°09″ (E), and altitude 111 m) during a period from 2018 to 2021 (Figure 1). A total of 100 maize samples from each year were taken evenly from the entire region of Northern Serbia. In Croatia 268 maize samples were analysed on DON, 191 samples on FUMs, 382 samples on ZEN, and 262 samples on T-2/HT-2 toxin concentrations. Samples were collected within the period 2018–2021 from different fields situated in four Croatian regions (Eastern, Central, Northern and Western Croatia), with latitude 45°10″ (N), longitude 15°30″ (E), and altitude 142 m (Figure 1). Since the largest production of maize is in Eastern Croatia, about 70% of the samples originate from that part of the country. Sampling in both countries was performed according to Commission Regulation (EC) No. 401/2006 [29], while in Serbia the Serbian Regulation [30] was also taken into account.

With the aim to examine the influence of weather conditions on the contamination of maize samples with *Fusarium* mycotoxins, and to avoid the possibility of secondary contamination, maize samples from both countries were collected during September and October (2018–2021), immediately after harvest or from dryers, before further storage in silo and distribution. 

In Serbia after harvest in each year, at the Institute of Food technology in Novi Sad, representative maize samples were prepared from approximately 10 kg of aggregate samples. Preparation of representative samples included: homogenization (Nauta mixer, model 19387, Nauta patenten, The Netherlands), quartering, milling (KnifetecTM 1095 mill, Foss, Hoganas, Sweden), packing in zip lock bags (150–200 g), and storage at −18 °C. The laboratory samples were removed from freezing at the beginning of 2022, and again homogenized (Rotary laboratory mixer RRM Mini-II, Ludwigshafen, Germany) before analysis on high-performance liquid chromatography-tandem mass spectrometry (LC-MS/MS). Before mycotoxins analysis in maize samples from Croatia by ELISA methods, each year, the prepared test portions were ground into a fine powder having a particle size of 1.0 mm using an analytical mill (Cylotec 1093, Tecator, Sweden), and then stored at 4 °C until analyses of particular mycotoxins within 48 h.

### 2.2. Fusarium Mycotoxins Analysis

In Serbia LC-MS/MS was used for analysis of the collected maize samples. The following chemicals were used: acetonitrile (ACN) (Fisher Scientific, Geel, Belgium) of HPLC grade and water (Fisher Scientific, Geel, Belgium), methanol (MeOH) (Carlo Erba, Val de Reuil, France), and formic acid (Fluka Analytical, Sigma Aldrich, Steinheim, Germany) of LC-MS/MS grade. 

Ultra-pure water was produced by Adrona Crystal EX HPLC Water Purification system (Riga, Latvia). Mycotoxin standards DON (100 μg/mL), FUMs (FB_1_ and FB_2_, each 50 μg/mL), HT-2 toxin (100 μg/mL), and ZEN (100 μg/mL) were purchased from Fluka Analytical (Steinheim, Germany), except for T-2 toxin (100 μg/mL) which was purchased from LGC Standards (Wesel, Germany). A mixed standard stock solution of 6 different mycotoxin standards was dissolved in MeOH/water (50:50, *v/v*). Correspondingly, matrix-matched standards were prepared by diluting an appropriate volume of the appropriate stock standard solution in the blank sample extract with MeOH/water (50:50, *v/v*), yielding concentration levels from 5 to 150 ng/mL for DON, from 0.5 to 200 ng/mL for FB_1_ and FB_2_, from 0.05 to 30 ng/mL for HT-2 toxin and T-2 toxin, and from 1 to 200 ng/mL for ZEN. Both mycotoxin standards and stock standard solutions were stored in the freezer at −18 °C until analysis.

Sample preparation and analysis were performed according to Application Note from Thermo Fisher Scientific [31]. Briefly, five grams of milled and homogenized sample was weighed into a 50 mL centrifuge tube. Twenty milliliters of ACN/water (80:20, *v/v*) extraction solvent was added and the tube was shaken on a horizontal shaker (6 Hz, 60 min). This was followed with centrifugation at room temperature (67 Hz, 5 min). Supernatant (400 μL) was diluted with 600 μL MeOH/water (50:50, *v/v*), and shaken on a vortex shaker. The supernatant was filtered through a 0.2 µm PTFE filter into HPLC vials and injected into the LC-MS/MS system. 

LC-MS/MS analysis was performed using an HPLC Vanquish Core system (Thermo Fisher Scientific, Waltham, MA, USA) equipped with a TSQ Quantis Triple Quadrupole mass spectrometer equipped with a heated electrospray ionization (HESI) source (Thermo Fisher Scientific, Waltham, MA, USA). A ZORBAX Eclipse Plus C18 column (2.1 × 100 mm, 1.8 µm) (Agilent, Santa Clara, CA, USA) was selected for chromatographic separation. A mobile phase consisting of water with 0.1% formic acid (mobile phase A) and 0.1% formic acid in methanol (mobile phase B) was used. The gradient elution program was as follows: 0 min 95% mobile phase A, 0.5 min 95% mobile phase A, 7 min 30% mobile phase A, 9 min 0% mobile phase A, 12 min 0% mobile phase A, 12.1 min 95% mobile phase A, and 15 min 95% mobile phase A. The mobile phase flow rate was 0.3 mL/min. The temperature of the column was set at 40 °C and the autosampler tray temperature was set at 20 °C. The sample injection volume was 10 µL. The mass spectrometer analyses were carried out using selected reaction monitoring (SRM) mode. The HESI source was operated under positive (3.5 kV) mode, except for ZEN in negative (2.5 kV) mode. The applied parameters were as follows: ion transfer tube, 325 °C; vaporizer temperature, 350 °C; cycle time, 0.5 s; collision induced dissociation gas pressure, 1.5 mTorr; auxiliary gas, 6 Arb; sheath gas, 30 Arb; and sweep gas, 1 Arb. Argon served as collision gas, while nitrogen served as auxiliary, sheath, and sweep gas. Data were acquired and analyzed using Thermo Scientific TraceFinder software TSQ Quantis 3.2 Tune (Thermo Fisher Scientific, Waltham, MA, USA).

The LC-MS/MS method was validated in accordance with the performance criteria outlined in Commission Decision [32] and Technical Report CEN/TR 16059:2010 [33]. The method was validated in terms of linearity, limit of quantification (LOQ), trueness, recovery, repeatability, and reproducibility. The validation study was conducted by the analysis of quality control materials (for DON, FB_1_, FB_2_, ZEN) and spiked uncontaminated maize samples (for T-2 and HT-2 toxin). The quality control materials, maize flours, for the examined mycotoxins were provided by: Trilogy analytical laboratory for FUMs (product code TR-F100; contained 2400 µg/kg of FUMs) and ZEN (product code TR-Z100; contained 59.4 µg/kg of ZEN), and Food Analysis Performance Assessment Scheme (FAPAS) for DON (Food Chemistry Proficiency Test 04378; contained 1200 µg/kg of DON). Matrix effects were accounted by using matrix matched calibration (MMC). The matrix effects were expressed as the signal suppression/enhancement (SSE) and calculated from the slope ratio for MMC and solvent calibration. For each examined *Fusarium* mycotoxin, the primary product ion which corresponds to the most abundant product ion was used for quantification, while the other two were used for confirmation: DON (249.0, 231.0, and 175.0), FB_1_ (334.1, 352.1, and 704.4), FB_2_ (336.1, 354.2, and 688.5), ZEN (175.0, 131.0, and 273.0), T-2 (245.1, 327.0, and 387.1), and HT-2 toxin (345.1, 255.1, and 285.1). The retention times for the examined *Fusarium* mycotoxins were the following: DON, 1.05 min; FB_1_, 10.85 min; FB_2_, 11.50 min; ZEN, 11.66 min; T-2, 11.30 min; and HT-2, 10.86 min.

All obtained validation parameters, for the applied LC-MS/MS method in this study, fulfil the criteria given under the Regulation 2006/401/EC [29] and the Technical Report [33]. All mycotoxins were quantified using MMC curves, because for all investigated mycotoxins SSEs were higher than ±20%. For all curves the squared correlation coefficients (R^2^) were above 0.998. LOQs for DON, FB_1_, FB_2_, ZEN, T-2, and HT-2 toxin were 50.0, 5.0, 5.0, 10.0, 0.50, and 0.50 µg/kg, respectively. The analysis of quality control materials yielded values for trueness: DON (104%), FB_1_ (83%), FB_2_ (81%), ZEN (110%), while the analysis of spiked uncontaminated maize samples yielded recovery: T-2 (96%), HT-2 toxin (88%). Repeatability and reproducibility were calculated as relative standard deviations and none of them exceeded 20%. Therefore, all obtained values for trueness, recovery, repeatability, and reproducibility were in accordance to the criteria specified in used regulations [29,33].

In Croatia, concentrations of mycotoxins were determined using competitive ELISA Ridascreen^®^ test kits for DON (Art. No. R5906), ZEN (Art. No. R1401), FUMs (Art. No. R3401), and T-2/HT-2 toxin (Art. No. R3805). Analyses were performed completely as instructed by the kits manufacturer (R-Biopharm, Darmstadt, Germany). Each kit contains: a microtiter plate with 96 wells coated with antibodies; standard solutions containing different concentrations of mycotoxin standards: DON (0, 3.7, 11.1, 33.3, 100 μg/L); ZEN (0, 50, 150, 450, 1350, 4050 ng/L); FUM (0, 0.025, 0.074, 0.222, 0.666, 2 mg/L), and T2-HT2 (0, 1, 3, 6, 12, 36 μg/L); an enzyme conjugate; an anti-antibody; the substrate and chromogen solution (urea peroxide/tetramethylbenzidine); washing and dilution buffers and stop solution. All other chemicals used in the analysis were of analytical grade. 

ELISA tests were evaluated using a ChemWell auto-analyzer (Awareness Technology Inc. 2910, Palm City, FL, USA) with the absorbance being measured at 450 nm. In order to determine mycotoxin concentrations in the sample, a standard curve was plotted based on the sample extract dilution factors. Implemented ELISA methods were validated in earlier studies [34,35,36,37] and final concentrations were calculated per each mycotoxin based on the average recoveries. Further, applied ELISA methods were validated as accredited in agreement with the ISO/IEC 17025 Standard [38]. LOQ values for DON, ZEN, FUMs, and T-2/HT-2 equaled to 22, 3, 24, and 5 µg/kg, respectively.

For all ELISA tests the validation was performed by determination of recovery and intermediate precision. The recovery rates were determined at three different levels (50, 100, and 200 mg/kg) by spiking the control maize samples with the standard in-house mycotoxin working solution (300 mg/L) corresponding to the assessed content levels. Regarding the determination of intermediate precision, the same steps were repeated on two other occasions by two different analysts and under the same analytical conditions. The mean recovery rates for DON, ZEN, FUMs, and T-2/HT-2 toxins were 98.2%, 89.2%, 75.2%, and 97.6%, while for intermediate precision the mean rates equaled to 95.9%, 86.8%, 72.1%, and 92.9%, respectively.

Quality control was performed by analysis of different reference materials (RMs) in parallel with each batch of the studied samples, so as to check whether the obtained concentration falls within the assigned range. The producer (FAPAS, Sand Hutton, York, UK) assigned ranges of RMs were: T04354QC, maize—1104 μg/kg (756–1452 μg/kg) for DON and 90.7 μg/kg (50.8–130.5 μg/kg) for ZEN; TYG079RM, maize flour—854 μg/kg (791–917 μg/kg) for FUM; and TYG087RM, cereal-based animal feed—523 μg/kg (496–550 μg/kg) for T-2/HT-2. Mycotoxin concentrations obtained with RMs were compared to the assigned values given by the manufacturer and were found to be within the defined ranges.

### 2.3. Weather Analysis

With the aim to investigate the influence of weather conditions on the natural occurrence of DON, FUMs, ZEN, T-2, and HT-2 toxin, a detailed analysis of weather condition parameters for maize growing seasons (April–September) in a period of four years (2018–2021) for investigated regions in Serbia and Croatia, was conducted. Weather condition parameters related to the monthly average air temperature and sum of precipitation, as well as drought indicators (Standardized Precipitation Index (SPI-2), Palmer Z Drought Index, and Palmer Drought Severity Index (PDSI)) were acquired from the Republic Hydrometeorological Service of Serbia [39] and from Croatian Meteorological and Hydrological Service [40]. Deviations of mentioned data were determined in comparison to the data recorded in the long-term period, from 1981 to 2010. 

### 2.4. Statistical Analysis

For statistical analysis of data obtained in the validation study, as well as for the analysis of mycotoxin occurrence, and weather condition parameters, Microsoft Excel 2010 was used. The following functions have been used: percentage, minimum, maximum, mean, median, standard deviation, and sum. The statistical analysis of data was performed only on positive samples in which the determined concentrations were higher than the LOQs of methods applied.

## 3. Results and Discussion

In this study, the natural occurrence of DON, FUMs, ZEN, T-2, and HT-2 toxins was examined in Serbian and Croatian maize samples collected over a four-year period (2018–2021), and the obtained results are interpreted in relation to the recorded weather data.

### 3.1. Serbia

#### 3.1.1. *Fusarium* Mycotoxins Occurrence in Serbia in the Period 2018–2021

Among 400 analyzed maize samples (100 samples each year), the overall average incidence of *Fusarium* mycotoxins in the investigated four-year period was as follows: 10% of maize samples contained DON, 97% FB_1_, 97% FB_2_, 1% ZEN, and 29% T-2 toxin, while 8% of samples contained HT-2 toxin. The frequency and the level of contamination of examined *Fusarium* mycotoxins varied by production year, as can be seen in Table 1.

Among examined mycotoxins in this study, FUMs (FB_1_ and FB_2_) were the most prevalent in each year of investigation. FB_1_ and FB_2_ contaminated 100% of the analyzed maize samples from years 2018, 2020, and 2021, while 89% and 88% of analyzed maize samples from year 2019 were contaminated with FB_1_ and FB_2_, respectively. The highest mean and median concentration of FB_1_ and FB_2_ were detected in maize samples from the year 2021 (Table 1). The second most common mycotoxin which occurred in Serbian maize samples was T-2 toxin, with the highest incidence (66%) in the year 2019, but with a very low average and median concentration (Table 1). In regard to DON and HT-2 toxin, it could be observed that those mycotoxins occurred in maize samples collected from the investigated period in the range from 5% to 17% and from 6% to 12%, respectively. Further, ZEN was the least frequently detected among examined *Fusarium* mycotoxins in this study. In the maize growing season 2019, none of the analyzed samples was contaminated with this mycotoxin, while in the other three years, the presence of ZEN was determined only between 1% to 2% of analyzed maize samples. From Table 1 it could be noted that the obtained concentrations of DON, ZEN, T-2, and HT-2 toxins in examined maize samples from Serbia did not exceed the maximum levels (MLs) intended for human or animal nutrition as regulated by both the European Union and Serbian Regulative [23,41,42,43]. Conversely, in examined maize samples from the four-year period, the sum of FB_1_ and FB_2_ concentrations exceeded ML of 4000 µg/kg in 16%, making them unsuitable for human nutrition, while none of the analyzed maize samples contained FUMs above the ML of 60,000 µg/kg intendent for animal nutrition. 

It is well known that the weather conditions (especially air temperature and amount of precipitation) during the maize growing season represent factors with the strongest influence on the occurrence of mycotoxins in maize in general [2]. Therefore, for a better interpretation of the obtained results in this study, an analysis of weather witnessed for the period 2018–2021 was undertaken.

#### 3.1.2. Weather in Serbia in 2018–2021

**Figure 2:**
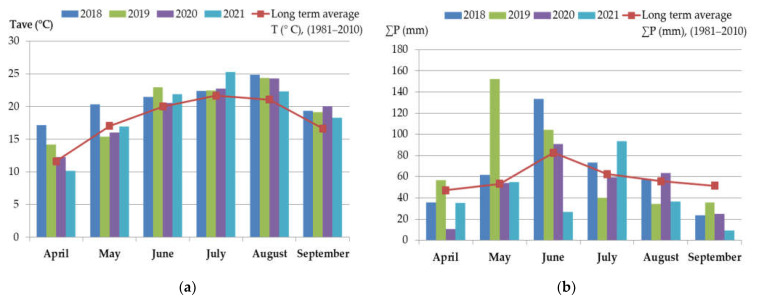
Monthly average (**a**) air temperatures and (**b**) sum of precipitation in Northern Serbia in the period 2018–2021 (April–September) in comparison with the long-term average values for the period 1981–2010.

The weather conditions (average air temperatures and sum of precipitation) compared to the long-term average values (1981–2010) during the maize growing season (April–September 2018–2021) in Northern Serbia are summarized and shown in Figure 2a,b. Additionally, drought indicators were analyzed for the period of the generative phase of maize from May to September (2018–2021), and are presented in Table 2.

It can be noticed (Figure 2a,b) that the monthly average air temperatures in each study year were mostly above the long-term average values (1981–2010) during the maize growing season. Only during April 2021 and May 2019, 2020, and 2021 were lower air temperatures recorded in comparison to the long-term average temperatures (1981–2010). On the other hand, considerable differences are observed in the sum of precipitation in the same period compared to long-term average values (Figure 2a,b). Namely, an extremely high amount of precipitation was recorded in May 2019 and June 2018, while in June 2021 a significantly lower sum of precipitation occurred. Further, the amount of precipitation during July was higher than the long-term average values in the 2018 and 2021 maize growing seasons, while in the other two years of investigation it was around (2020) or lower (2019) than the long-term average sum values (1981–2010). In all investigated years, air temperature considerably deviated in August in comparison to the long-term average, while the sum of average precipitation was around (2018, 2020) or lower (2019, 2021) than the long-term average. The air temperature was considerably higher for all years in September followed by a considerably lower sum of precipitation compared to the long-term average. 

To determine the dry/wet conditions during investigated years, values of three different drought indicators were considered (Table 2).

According to the SPI-2, which represents deviations of the observed total precipitation over a 2-month accumulation period, 2018 has been characterized mainly as normal except for May, which was characterized as moderately humid. Further, May and June in 2019 were extremely humid, while the other three months of the observed period were normal. In 2020 all investigated months were characterized as normal except May, which was characterized as severe drought. The year 2021 was characterized with normal (May, July, August) to moderate drought condition (June–September). Palmer Z Drought Index as a measure of short-term drought on a monthly scale indicated weather conditions from normal to extreme drought during a period of May–September in 2021, and from extremely humid to moderate drought in 2019 in the same period. In 2018 and 2019 (May–September), normal to severe drought and extremely humid to moderate drought, respectively, were recorded. Furthermore, the Palmer Drought Severity Index is a standardized index based on a simplified soil water balance; estimated relative soil moisture conditions indicate normal conditions during period May–September in 2018 and 2019, and dry weather conditions during 2020 and 2021, particularly in 2020. 

Briefly, the weather conditions in the period from April to September during the four-year (2018–2021) investigation were as follows: 2018 warmer and slightly wetter than average conditions; 2019 and 2020 warmer with normal humidity; and 2021 warmer and drier than average condition (Figure 2, Table 2). However, in 2021 climatic extremes were recorded, such as late frosts during the spring, drought during the summer months, and there were hailstorms and storms, which also had a negative impact on agricultural crop production [44]. Weather conditions in the investigated period (2018–2021), as can be seen from Table 2, did not prove to be suitable for the synthesis of most of the analyzed *Fusarium* mycotoxins, with the exception of FB_1_ and FB_2_. As already stated above, the highest mean concentration of FB_1_ (4684 ± 3517 µg/kg) and FB_2_ (1300 ± 994 µg/kg) and median concentration of FB_1_ (3921 µg/kg) and FB_2_ (1073 µg/kg) in 2021, were determined. Furthermore, in 2021 the highest quantified concentrations of FB_1_ and FB_2,_ 21,239 µg/kg and 5825 µg/kg, respectively, were observed. These data could be related to the amount of precipitation and the average daily temperature during the flowering period of maize. Namely, in July 2021, a considerably higher average amount of precipitation was recorded (93.6 mm), which represents 49% more precipitation compared to the long-term average (63 mm). The precipitation in July was often local and came in the form of rainstorms with a huge precipitation fall in a short amount of time. In the same month, deviation of the average daily temperature from the long-term average of 3.1 °C was recorded, while according to the drought indicators (SPI-2, Z, and PDSI index), normal weather conditions were registered (Figure 2a,b; Table 2). When we consider the values of the SPI-1, which represent deviations of the observed total precipitation over a 1-month accumulation period, July in 2021 was characterized with moderate humid conditions according the SPI-1 Index (1.0).

Considering all mentioned facts, high average daily temperatures, high sum of precipitation, and moderate humid weather conditions during July in 2021 were favorable for infection and growth of *Fusarium* species; as reported worldwide, FUMs are a group of mycotoxins mainly produced by *F. verticillioides* and *F. proliferatum* under favorable high-temperature and humid climates [45]. Furthermore, the lack of precipitation and higher average mean daily temperatures during August and September in 2021 led to FUMs synthesis in high concentrations, as a response of *Fusarium* species to the unfavorable weather conditions. This conclusion is based on the results of a study conducted by Marin et al. [46]. Namely, in their investigation [46], the effects of ecophysiological factors, temperature, and solute potential on both growth and the regulation of the FUMs biosynthetic FUM1 gene were studied and compared in one isolate of each of the two closely related FUMs-producing maize pathogens *F. verticillioides* and *F. proliferatum.* Both of the investigated *Fusarium* species have shown similar profiles of growth (17–35 °C), but optimal growth condition for *F. verticillioides* was maintained at higher temperatures and lower solute potential values. The results of this study indicated that environmental conditions leading to water stress (drought) might result in increased risk of FUMs contamination of maize caused by *F. verticillioides.* On the other hand, *F. proliferatum* showed a stable expression pattern of the FUM1 gene regardless of water potential conditions [46]. It could be assumed that both *F. verticillioides* and *F. proliferatum* occurred in Serbian maize, since FUMs were present in very high frequency in the maize samples from all four investigated growing seasons (2018–2021).

### 3.2. Croatia 

#### 3.2.1. Fusarium Mycotoxins Occurrence in Croatia in the Period 2018–2021

The frequency and the contamination level of maize with *Fusarium* mycotoxins determined in Croatia during the period 2018–2021 are shown in Table 3. The overall average incidence of *Fusarium* mycotoxins in maize for the investigated four-year period showed that 78% of maize samples are contaminated with DON, 89% with FUMs, 45% with ZEN, and 50% with T-2/HT-2 toxin. 

In Croatia during the period 2018–2021, the highest occurrence for DON (95%) was observed in 2021 and its highest mean concentration was observed in 2020 (922 ± 1511 μg/kg). Occurrence for ZEN (63%) and FUMs (95%) was the highest in 2020, but contrary to DON, the highest mean concentrations for these mycotoxins were determined in 2021 (ZEN 182 ± 242 μg/kg and FUMs 1314 ± 1740 μg/kg). Values higher than MLs defined for maize as food were determined in 4% of the samples analyzed for DON, 3% for FUMs, and 0.5% for ZEN, whereas for T-2/HT-2 toxin 1% of the samples was not in accordance with recommended values defined for maize as food. However, according to the obtained concentrations for all four analyzed mycotoxins, all samples were compliant to be used as feed. In order to connect the results of mycotoxin occurrence and concentrations obtained in this study, with the weather witnessed for the period 2018–2021 in Croatia, those data were further presented and interpreted.

#### 3.2.2. Weather in Croatia in the Period 2018–2021

**Figure 3:**
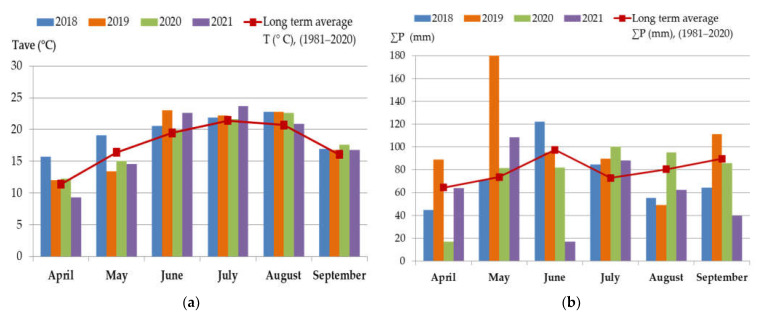
Monthly average (**a**) air temperatures and (**b**) sum of precipitation in Croatia in the period 2018–2021 (April–September) in comparison with the long-term average values for the period 1981–2010.

The average air temperatures and sum of precipitation in the period April–September 2018–2021 compared to the long-term average values in the period 1981–2010 during the maize growing season in Croatia are shown in Figure 3a,b. Additionally, drought indicators for the period from June to August (2018–2021), are presented in Table 4. 

Analysis of meteorological data from stations in the lowland part of Croatia indicates lower air temperatures during May (2019, 2020, and 2021) when booting of maize usually occurs. In addition to lower air temperatures, an extremely high amount of precipitation was recorded during May of 2019. The amount of precipitation also increased during May 2021, while in May of 2018 and 2020 precipitation did not deviate considerably from the long-term average of 1981–2010. The amount of precipitation during July, when the flowering of maize mainly takes place, was higher than the long-term average in all analyzed years. Air temperature was considerably higher in July of 2021, while during July of 2018, 2019, and 2020, air temperature did not deviate considerably from the long-term average. August of 2018, 2019, and 2020 was warmer in comparison to the long-term average, while the total amount of precipitation recorded during August of 2018, 2019, and 2021 was lower than the long-term average.

Values of three different drought indicators were examined to determine the dry/wet conditions during the analyzed years (Table 4).

The SPI-2 index shows deviations of the observed total precipitation over a 2-month accumulation period. The Palmer Z index was calculated on a monthly basis, while the PDSI carries information about long-term drought. According to the SPI-2 and Z drought indicators, wet conditions occurred during June 2019, while the Z drought indicator indicates slightly wet conditions in June 2021 as well. The PDSI indicator indicates dry conditions during 2018, 2019, and 2020.

It is known that humid and cool conditions, in addition to moderate temperatures (between 20 and 30 °C) during the period of maize booting, together with high relative humidity (90%), frequent rainfall during and after flowering, extended periods of high moisture, and the occurrence of air currents promote *Fusarium* spp. development. Data also suggests that precipitation levels influence fungi and mycotoxin synthesis to a greater extent compared to the influence of temperature [47,48]. Taking into account drought indicators registered in Croatia for the period May-September (Table 4), results of this study determined for DON, ZEN, and FUM in general can be put into connection with the higher amount of precipitation in May 2019 and 2021 in comparison to the long-term average for this month. Lower contamination in 2018 can be put into connection with data which shows that May of 2018 did not deviate considerably from the long-term average. Additionally, data show that August 2020 was slightly rainier than average. For other monthly average indicators of air temperatures and sum of precipitation for months of the period 2018–2021 in Croatia, there is no clear link with mycotoxin contamination. The highest prevalence of T-2/HT-2 toxin was determined in 2019 (84%), which can be explained with a very to extremely wet June 2019, which followed an extremely wet May 2019. However, due to the interaction of many various factors that may affect the biosynthesis of *Fusarium* mycotoxins during maize cultivation, such as genetic factors, mechanical damage of kernels, pest infestation, mineral plant nutrition, poor harvest and storage practices, and/or chemical treatment, as observed in some earlier studies [49], the contamination observed in this study also cannot be put into relation solely to the weather.

### 3.3. Comparison of Fusarium Mycotoxins Occurrence for the Period 2012–2021

**Figure 4:**
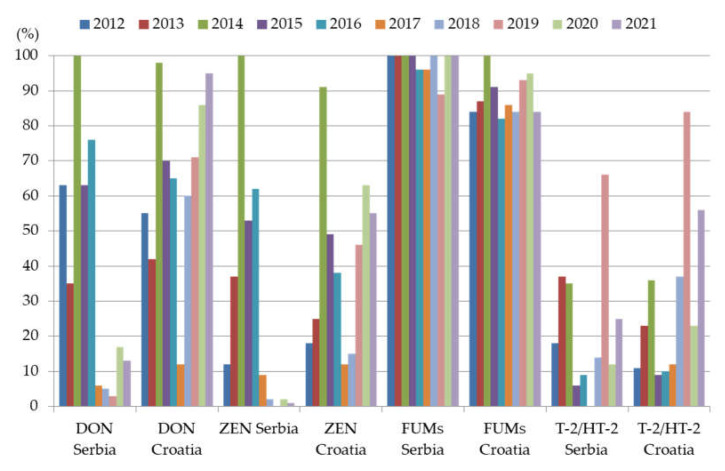
An overview of *Fusarium* mycotoxins occurrence in maize from Serbia and Croatia in the period 2012–2021.

Taking into account the period of ten years (2012–2021), it can be observed that investigated *Fusarium* mycotoxins occurred very frequently in maize cultured in Serbia and Croatia (Figure 4). Figure 4 shows an overview of the occurrence data for DON, ZEN, FUMs, and T-2/HT-2 toxins, represented as a percentage of contaminated samples. In Figure 4, published data for maize growing seasons 2012–2015 [50] and 2016–2017 [51] for Serbia were summarized. For Croatia, data were partially published [37,40,41,42], while the other data were not published. Earlier unpublished data and data obtained in this study are also included in Figure 4. The same sites and methodology of sampling were applied as described for the period 2018–2021 for both countries. Further, in Croatia, all mycotoxin analyses, in the period 2012–2017, were conducted by the ELISA methods as described earlier in this study. Serbian maize samples for the period 2012–2015 and 2016–2017 were analyzed by the validated LC-MS/MS methods described in detail by Malachová et al. [52] and Sulyok et al. [53], respectively.

Merged data for Serbia, presented in Figure 4, obtained in our previous studies [49,50] investigated the contamination of maize with *Fusarium* mycotoxins in the period 2012–2017. The drought indicators for the period 2012–2017 are given in the Table 5, which describes the summer months of the investigated years in our previous studies. 

In these studies, the examined period of six years included maize-growing seasons with extreme drought (2012), hot and dry conditions (2013, 2015, and 2017), extreme rainfall (2014), and weather without significant deviations (2016) compared to the long-term period (Table 5). The results showed that FB_1_ and FB_2_, regardless of considerable differences in recorded weather conditions, occur in maize samples with a very high frequency of contamination, most often between 96–100%. However, the highest concentrations of FB_1_ (27,103 µg/kg) and FB_2_ (4651 µg/kg) were determined in maize samples collected in 2014, in which the maximum recorded value of precipitation registered occurred, since the beginning of meteorological observations in Serbia. In terms of DON and ZEN, the obtained results indicate significant differences in their occurrence in maize samples collected over the period of six years. Both mycotoxins occurred with a prevalence of 100% in maize samples collected from 2014, which was characterized as an extremely rainy year. DON was detected in the concentration range from 428 to 16,350 µg/kg, and even in 84% of samples its concentrations were higher than the ML of 1750 µg/kg defined for maize in food. Further, ZEN was also detected in the highest concentrations (15–2596 µg/kg) in maize from 2014, while in 51% of samples its concentration exceeded ML (350 µg/kg) for maize in food. In less than, or around 10% of samples, detected concentrations of both DON and ZEN were higher than the ML stipulated for maize intended to be used as a feed material. Among six investigated years, in 2014 DON and ZEN were detected in maize with the highest concentrations as well as contamination frequency. In the other five examined years, DON (6–76%) and ZEN (9–62%) occurred with different contamination frequency, while their determined concentrations were lower than MLs for maize intended for food and feed. T-2 and HT-2 contaminated between 6–66% and 12–16%, respectively, of maize samples examined in the period 2012–2017, while no considerable differences were found between their concentrations determined in maize samples originated from different years.

Results of earlier studies concluded that maize is one of the most frequent crops in Croatia that is often contaminated with *Fusarium* mycotoxins [34,35]. As in Serbia, studies performed in the last period in Croatia showed huge variations of their concentrations, with high incidence influenced by environmental factors such as temperature, humidity, drought, and rainfall during pre-harvest and harvest periods. High concentrations of these mycotoxins could be linked to frequent rainfalls, low temperatures, and significantly lower than average temperatures in the period of maize growth, which increased the contamination of maize with *Fusarium* moulds and production of their secondary metabolites. The drought indicators for the period 2012–2017 in Croatia are given in the Table 6.

Moderate occurrence of all investigated *Fusarium* mycotoxins in Croatia during 2012 and 2013 (unpublished data) can be connected with normal to mild or extreme drought indicators registered during the period of maize growth to harvest. Higher mycotoxin concentrations determined in Croatia during 2014 can be linked with moderate humid drought indicators. These indicators in 2015 were found to be normal to mild drought or mild humid during the period of June to September. Furthermore, generally lower maize contamination with *Fusarium* mycotoxins determined in 2016 and 2017 (unpublished data), especially lower in comparison to the most contamination in 2014, can be put into connection with normal drought indicators during the same observed period.

Concentrations of DON determined in Croatia in 2014 were higher than MLs [23] in 27% of maize samples, while in 2015 in 44% of maize samples [36]. Levels higher than the guidance values given for feedstuffs (2006/576/EC) were observed in 3% (in 2014) and 7% (in 2015) of maize samples. The highest mean DON concentration of 1998 ± 2517 µg/kg established in 2014 and 3711 ± 2710 µg/kg established in 2015 were determined in maize. Maximal DON levels observed in 2014 and 2015 were 9270 µg/kg and 9560 µg/kg, respectively. The mean ZEN concentration determined in 2014 was 810 ± 858 µg/kg, in 2015 the concentration was 1519 ± 1754 µg/kg, the maximal ZEN concentration in 2014 was 3217 µg/kg, and in 2015 it was 7874 µg/kg. Kiš et al. [49] analysed T-2/HT-2 toxin in maize from different fields located in three Croatian regions during 2017–2018. The highest mean sum of concentration in maize was 54 ± 85 µg/kg. In two maize samples, sum concentrations of T-2/HT-2 toxin were higher (332 µg/kg and 253 µg/kg, respectively) than the indicative level stipulated for maize (200 µg/kg). Authors linked obtained results to substantial temperature variations and high precipitation seen during the maize growth and harvesting periods. 

In regard to both countries, as can be seen from Figure 4, the greatest prevalence of DON and ZEN among the investigated period of 10 years (2012–2021) was noticed in maize samples collected in 2014. The authors of the previously reported studies [50,51] indicated that extremely high amounts of precipitation in 2014 influenced 96% and 100% of maize samples from Serbia, and 98% and 91% of samples contaminated with DON and ZEN, respectively. As can be seen from Figure 4, the percentage of contaminated maize samples with DON in the period from 2017 to 2021 was low (6–13%) in Serbia, while Croatian maize samples were contaminated with higher frequency in the same period (12–95%). Further, in terms of ZEN, it was also detected with lower frequency in Serbia (0–9%) in comparison to Croatia (12–63%) in the period from 2017 to 2021. Regarding prevalence of the sum of T-2 and HT-2 toxins, it can be noted that in Croatian maize they occurred more frequently (9–84%) than in Serbian maize (0–66%) in the investigated ten year-period. The highest percentage of contaminated maize with T-2 and HT-2 toxins in both countries was recorded in 2019, which was characterized with a higher amount of sum of precipitation in the period May-August. In the investigated ten year-period, FUMs were the most prevalent *Fusarium* mycotoxins in Serbian maize, since in 7 of 10 investigated years, 100% of the examined samples were contaminated with FB_1_ and FB_2_. As in Serbia, FUMs in maize from Croatia occurred with the highest prevalence during the investigated ten year-period (82–100%). 

Based on the obtained results in this study, as well as the results of our previous study, it can be noticed that in the period of ten years (2012–2021), investigated *Fusarium* mycotoxins occurred very frequently in maize from Serbia and Croatia. Changes in weather conditions, especially increased amounts of precipitation, influenced the increased prevalence of certain *Fusarium* mycotoxins, particularly DON and ZEN. Contrary to this, FUMs occurred with high frequency in maize from each of the ten investigated years. It could also be observed that maize samples from each year in the period 2012–2021 represent a mixture of *Fusarium* mycotoxins. This fact should be taken into consideration, since a co-occurrence of different mycotoxins may generate synergistic or additive effects in humans and animals [54].

The climate of Serbia and Croatia is described as a moderate continental climate. However, in recent years, from 2012 to the present, changes in weather conditions followed by dry and hot conditions with occasional extreme precipitation have been recorded. It is obvious that the trend of increasing frequency of extreme weather events and a changing climate is becoming more and more noticeable. All of the mentioned above has already had an impact on the occurrence of *Fusarium* mycotoxins in maize from Serbia and Croatia. In both Serbia and Croatia, in the last 10 years, FUMs became the most prevalent *Fusarium* mycotoxins in cultivated maize. On the other side, changes in weather events in the period 2012–2021, especially dry and hot conditions in 2012, 2013, 2015, 2017, and 2021 in both countries, influenced aflatoxin contamination of cultivated maize [55]. It is in line with recent quantitative estimations [56,57] which have shown that in certain regions of Europe, increased DON (in wheat) and aflatoxin B1 (in maize) contaminations are expected, as a result of global warming and changeable weather events.

## 4. Conclusions

The frequency and contamination level of *Fusarium* mycotoxins examined in this study during the period 2018–2022 are shown to vary by year of maize production, and could be linked to climatic conditions per investigated country. Among them, FUMs were found to be the most common contaminants of maize in both Serbia and Croatia. The results indicate that dry and hot weather witnessed in the year 2021 resulted in the highest mean content of FUMs (5984 ± 4500 µg/kg) in maize samples in Serbia, while in Croatia the highest mean content of FUMs (1371 ± 2235 µg/kg) was observed in 2019. Considering the period of 2012–2022, the highest concentrations of *Fusarium* mycotoxins, especially DON and ZEN, were determined in maize samples cultivated in 2014, which can be liked to an extreme level of precipitation observed for that year in both countries, whereas FUMs occurred with high prevalence in maize from each of the ten investigated years. However, due to the interaction of many various factors that may affect the biosynthesis of *Fusarium* mycotoxins during maize cultivation, the contamination observed in this study cannot be put into relation solely to the climate conditions. Therefore, further research is needed on the influence of numerous meteorological and agrotechnical factors on the occurrence of mycotoxins in maize, as well as in other cereals.

## Figures and Tables

**Table 1:** *Fusarium* mycotoxins occurrence in maize from Serbia in the period 2018–2021.

Mycotoxin	Year	N (%) ^1^	Min–Max (µg/kg) ^2^	Mean ± Sdv (µg/kg) ^3^	Median (µg/kg) ^4^
DON	2018	5 (5)	50–148	85 ± 43	60
	2019	3 (3)	50–277	160 ± 114	152
	2020	17 (17)	53–392	144 ± 82	123
	2021	13 (13)	56–752	162 ± 181	139
FB_1_	2018	100 (100)	19–4403	978 ± 732	830
	2019	89 (89)	9–3793	614 ± 663	382
	2020	100 (100)	45–2435	739 ± 583	533
	2021	100 (100)	401–21,239	4684 ± 3517	3921
FB_2_	2018	100 (100)	17–1297	268 ± 206	221
	2019	88 (88)	5–1143	171 ± 192	100
	2020	100 (100)	12–638	186 ± 155	136
	2021	100 (100)	120–5825	1300 ± 994	1073
ZEN	2018	2 (2)	17–43	30 ± 19	30
	2019	nd ^5^	nd	nd	nd
	2020	2 (2)	17–57	37 ± 28	37
	2021	1 (1)	27	nd	nd
T-2 toxin	2018	14 (14)	0.5–24	5 ± 7	2
	2019	66 (66)	0.7–17	3 ± 3	3
	2020	12 (12)	0.6–6	2 ± 2	1
	2021	25 (25)	0.5–62	7 ± 12	3
HT-2 toxin	2018	6 (6)	1.5–32	13 ± 12	7
	2019	9 (9)	0.6–24	6 ± 8	1
	2020	6 (6)	0.6–13	6 ± 4	6
	2021	12 (12)	0.7–36	8 ± 11	2

^1^ N (%): number (percentage) of contaminated samples; ^2^ Min-Max: minimum and maximum concentrations (µg/kg); ^3^ Mean ± Std: mean concentration (µg/kg) ± standard deviation (µg/kg); ^4^ Median: median concentration (µg/kg); ^5^ nd: not detected, i.e., below the limit of quantification (LOQ).

**Table 2:** Drought indicators registered in Serbia in May-September in the period 2018–2021.

Month	Year							
	2018		2019		2020		2021	
	Standardized Precipitation Index for 60 days (SPI-2)							
May	−0.3	N ^1^	1.9	EH ^2^	−1.4	SD ^3^	−0.1	N
June	−0.3	N	1.7	EH	0.3	N	−1.0	MoD ^4^
July	1.2	MoH ^5^	−0.2	N	0.6	N	−0.1	N
August	0.4	N	−0.3	N	0.8	N	0.6	N
September	−0.6	N	−0.3	N	0.0	N	−1.2	MoD
	Palmer Z Drought Index (Z)							
May	−1.2	N	3.6	EH	−1.6	MoD	0.2	N
June	−0.4	N	1.1	MoH	1.3	MoH	−3.9	ED ^6^
July	0.7	N	−1.7	MoD	−0.3	N	0.5	N
August	−0.8	MoD	−1.6	MoD	0.4	N	−1.1	MoD
September	−2.3	SD	−1.0	N	−2.8	ED	−2.8	ED
	Palmer Drought Severity Index (PDSI)							
May	−0.7	N	−0.9	N	−3.9	SD	−0.4	N
June	−0.6	N	0.4	N	−3.0	SD ^3^	−2.2	MoD
July	0.7	N	−0.7	N	−2.6	MoD	−1.6	N
August	0.1	N	−1.4	N	−2.0	MoD	−2.0	MoD
September	−1.6	N	−1.9	N	−2.9	MoD	−2.8	MoD

^1^ N: normal; ^2^ EH: extremely humid; ^3^ SD: severe drought; ^4^ MoD: moderate drought; ^5^ MoH: moderate humid; ^6^ ED: extreme drought.

**Table 3:** *Fusarium* mycotoxins occurrence in Croatia in the period 2018–2021.

Mycotoxin	Year	N ^1^	N (%) ^2^	Min–Max (µg/kg) ^3^	Mean ± Sdv (µg/kg) ^4^	Median (µg/kg) ^5^
DON	2018	78	47 (60)	22–1232	235 ± 324	104
	2019	72	51 (71)	27–4688	473 ± 862	168
	2020	56	48 (86)	93–9923	922 ± 1511	493
	2021	62	59 (95)	43–5134	875 ± 924	572
FUMs	2018	61	51 (84)	28–13800	1006 ± 2170	336
	2019	61	57 (93)	30–11530	1371 ± 2235	504
	2020	37	35 (95)	24–5920	962 ± 1453	476
	2021	32	27 (84)	61–6330	1314 ± 1740	398
ZEN	2018	99	15 (15)	3.6–479	76 ± 125	23
	2019	114	53 (46)	3.1–658	69 ± 146	9
	2020	84	53 (63)	3.1–1241	120 ± 214	26
	2021	85	47 (55)	8.2–1170	182 ± 242	95
T-2/HT-2	2018	93	34 (37)	11–332	58 ± 73	36
	2019	94	79 (84)	10–283	32 ± 40	18
	2020	39	9 (23)	11–42	18 ± 10	13
	2021	36	20 (56)	11–407	56 ± 93	25

^1^ N: number of total samples; ^2^ N (%): number (percentage) of contaminated samples; ^3^ Min–Max: minimum and maximum concentrations (µg/kg); ^4^ Mean ± Std: mean concentration (µg/kg) ± standard deviation (µg/kg); ^5^ Median: median concentration (µg/kg).

**Table 4:** Drought indicators registered in Croatia for May–September in the period 2018–2021.

Month	Year							
	2018		2019		2020		2021	
	Standardized Precipitation Index for 60 days (SPI-2)							
May	−0.4	N ^1^	1.9	EW ^2^	−0.7	N	0.7	N
June	0.5	N	1.5	VW ^3^	0.0	N	−0.8	N
July	0.7	N	0.3	N	0.2	N	−1.3	SD ^4^
August	−0.1	N	−0.1	N	0.7	N	0.1	N
September	−0.6	N	0.0	N	0.3	N	−0.9	N
	Palmer Z Drought Index (Z)							
May	−1.5	MoD ^5^	−0.1	N	−3.2	ED ^6^	0.1	N
June	−0.4	N	4.1	EW	−0.1	N	1.5	SW ^7^
July	0.8	N	−0.6	N	−1.0	MoD	−3.5	ED
August	0.6	N	0.5	N	0.5	N	−0.1	N
September	−1.0	MoD	−1.3	MoD	0.2	N	−1.1	MoD
	Palmer Drought Severity Index (PDSI)							
May	0.4	N	−1.0	N	−2.2	MoD	−0.6	N
June	0.4	N	−1.1	MiD ^8^	−2.3	MoD	−1.8	MiD
July	0.5	N	−1.0	N	−1.8	MiD	−1.7	MiD
August	−0.5	N	−1.3	MiD	−1.6	MiD	−1.9	MiD
September	−0.8	N	−1.1	MiD	−1.7	MiD	−2.2	MoD

^1^ N: normal; ^2^ EW: extremely wet; ^3^ VW: very wet; ^4^ SD: severe drought; ^5^ MoD: moderate drought; ^6^ ED: extreme drought; ^7^ SW: slightly wet, ^8^ MiD: mild drought.

**Table 5:** Drought indicators registered in Serbia in June–September in the period 2012–2017.

Month	Year											
	2012		2013		2014		2015		2016		2017	
	Standardized Precipitation Index for 60 days (SPI-2)											
June	−0.8	N ^1^	0.6	N	1.0	MoH ^2^	−0.3	N	1.0	MoH	−0.6	N
July	−1.1	MoD ^3^	−0.9	N	0.7	N	−2.7	ExD ^4^	0.9	N	−1.3	SD ^5^
August	−0.8	N	−0.6	N	1.2	MoH	−0.4	N	0.5	N	−0.8	N
September	−2.1	ED	0.4	N	1.3	MoH	0.6	N	0.4	N	0.0	N
	Palmer Z Drought Index (Z)											
June	−3.9	ED ^6^	−0.5	N	−1.5	SD	−2.8	ED	1.8	MoH	−3.1	ED
July	−1.4	MoD	−2.4	SD	2.9	VH ^7^	−3.9	ED	0.0	N	−3.1	ED
August	−3.9	ED	−1.4	MoD	1.2	MoH	0.2	N	0.5	N	−2.3	SD
September	−0.7	MoD	0.8	N	3.9	EH ^8^	−0.1	N	0.0	N	0.1	N
	Palmer Drought Severity Index (PDSI)											
June	−3.9	SD	1.1	N	−0.9	N	0.4	N	1.4	N	−2.9	MoD
July	−4.1	ED	−0.1	N	1.6	MoH	−2.8	MoD	1.3	N	−3.6	SD
August	−4.9	ED	−2.0	SD	1.9	MoH	−2.4	MoD	1.3	N	−4.0	ED
September	−4.0	ED	−0.9	N	3.4	VH	−2.0	MoD	1.0	N	−3.6	SD

^1^ N: normal; ^2^ MoH: moderate humid; ^3^ MoD: moderate drought; ^4^ ExD: exceptional drought; ^5^ SD: severe drought; ^6^ ED: extreme drought; ^7^ VH: very humid; ^8^ EH: extremely humid.

**Table 6:** Drought indicators registered in Croatia in June–September in the period 2012–2017.

Month	Year											
	2012		2013		2014		2015		2016		2017	
	Standardized Precipitation Index for 60 days (SPI-2)											
June	0.4	N ^1^	−0.3	N	1.1	MoH ^2^	0.7	N	0.5	N	−0.8	N
July	−0.7	N	−1.1	MoD ^3^	0.8	N	−1.5	SD ^4^	0.7	N	−0.8	N
August	−2.2	ED ^5^	−0.3	N	1.4	MoH	−0.4	N	0.4	N	−0.7	N
September	−1.1	MoD	0.4	N	1.8	MoH	0.1	N	−0.5	N	0.5	N
	Palmer Z Drought Index (Z)											
June	−0.2	N	0.8	N	3.1	SH ^6^	2.9	SH	0.7	N	−0.6	N
July	−1.6	MiD ^7^	−1.4	MiD	−0.1	N	−1.8	MiD	0.5	N	−2.1	MoD
August	−3.0	SD	−1.1	MiD	2.4	MoH	−1.5	MiD	1.3	MiH ^8^	−1.4	MiD
September	−4.2	ED	−0.8	N	2.3	MoH	−0.6	N	−0.1	N	−2.5	MoD
	Palmer Drought Severity Index (PDSI)											
June	−5.1	ED	−0.3	N	1.4	MiH	2.1	MoH	1.9	MiH	−0.8	N
July	−5.4	ED	−0.6	N	1.9	MiH	1.7	MiH	1.4	MiH	−1.0	N
August	−6.0	ED	−0.7	N	2.3	MoH	1.5	MiH	1.0	N	−1.4	MiD
September	−5.5	ED	−0.6	N	3.3	SH	1.4	MiH	0.6	N	0.0	N

^1^ N: normal; ^2^ MH: moderate humid; ^3^ MoD: moderate drought; ^4^ SD: severe drought; ^5^ ED: extreme drought; ^6^ SH: severe humid; ^7^ MiD: mild drought, ^8^ MiH: mild humid.

## Data Availability

Data is contained within the article.
